# A machine learning artefact detection method for single-channel infant event-related potential studies

**DOI:** 10.1088/1741-2552/ad5c04

**Published:** 2024-07-16

**Authors:** Simon Marchant, Marianne van der Vaart, Kirubin Pillay, Luke Baxter, Aomesh Bhatt, Sean Fitzgibbon, Caroline Hartley, Rebeccah Slater

**Affiliations:** 1 Department of Paediatrics, University of Oxford, Oxford, United Kingdom; 2 FMRIB, Nuffield Department of Clinical Neurosciences, University of Oxford, Oxford, United Kingdom

**Keywords:** machine learning, random forest, artifacts, electroencephalography, infant, evoked potentials

## Abstract

*Objective*. Automated detection of artefact in stimulus-evoked electroencephalographic (EEG) data recorded in neonates will improve the reproducibility and speed of analysis in clinical research compared with manual identification of artefact. Some studies use very short, single-channel epochs of EEG data with little recorded EEG per infant—for example because the clinical vulnerability of the infants limits access for recording. Current artefact-detection methods that perform well on adult data and resting-state and multi-channel data in infants are not suitable for this application. The aim of this study was to create and test an automated method of detecting artefact in single-channel 1500 ms epochs of infant EEG. *Approach*. A total of 410 epochs of EEG were used, collected from 160 infants of 28–43 weeks postmenstrual age. This dataset—which was balanced to include epochs of background activity and responses to visual, auditory, tactile and noxious stimuli—was presented to seven independent raters, who independently labelled the epochs according to whether or not they were able to visually identify artefacts. The data was split into a training set (340 epochs) and an independent test set (70 epochs). A random forest model was trained to identify epochs as either artefact or not artefact. *Main results*. This model performs well, achieving a balanced accuracy of 0.81, which is as good as manual review of data. Accuracy was not significantly related to the infant age or type of stimulus. *Significance*. This method provides an objective tool for automated artefact rejection for short epoch, single-channel EEG in neonates and could increase the utility of EEG in neonates in both the clinical and research setting.

## 1. Introduction

Electroencephalography (EEG) is a safe, non-invasive method of detecting and recording brain activity. It is commonly used both clinically and in a research context for assessing responses to sensory stimulation and for measuring background brain activity. A potential limitation of EEG is that the signals can frequently be contaminated by artefacts that can overwhelm the neurophysiological signal of interest [1]. These artefacts can arise from a variety of sources: examples include movement; detachment of the electrodes; electrical noise from inside or outside the body; and standing potentials and salt bridges on the scalp. Neonatal EEG, including stimulus-evoked EEG activity, is susceptible to movement artefact which can be difficult even for experienced users to accurately identify [2, 3]. This is of particular concern for studies of noxious stimuli, which evoke withdrawal reflexes [4] and may suffer from contamination by stimulus-correlated motion.

Identification of artefact in EEG in both the clinical and research setting is usually performed by expert human review of the EEG signals. This is time consuming, requires expert input, and reduces the reproducibility of artefact detection, introducing a potential source of variability in results. To address this problem, a number of software-based EEG artefact detection algorithms have been developed [5–11]. The most common artefact detection techniques used in these algorithms are independent component analysis (ICA) and artefact subspace reconstruction (ASR). ICA deconstructs the EEG signal into multiple component signals, based on the assumption that artefacts are generated independently from neural activity [12]. The obtained components (timeseries) are subsequently classified as being generated by a brain or non-brain source [13], using manual identification, thresholding [14] or supervised machine learning [15], and non-brain components are removed from the original EEG timeseries [13]. ASR uses principal component analysis to decompose a signal into components, and identifies those that differ to a clean calibration set [8]. It can separate no more components than there are channels, and so cannot be applied to single-channel data [16]. These methods are much less effective at reducing signal-to-noise ratio (SNR) with low numbers of channels [17] and require long duration recordings, either from a single long recording or multiple short recordings from the same subject and session [1]. Alternative methods have also been developed, for example using the extreme standardised deviate of the voltage amplitude to detect artefactual recordings [18]. Recently, there has also been interest in automated methods for artefact recognition in infants, using ICA [19, 20], ASR [21], and channel similarity [22]. Most recently, Hermans *et al* [23] developed a machine learning model using semi-supervised methods to identify artefact in neonatal EEG. These methods have similar limitations to the ICA approach, and are optimal for long duration recordings from multiple electrodes.

The acquisition of EEG data in neonates presents some specific data analysis challenges related to the detection of artefacts. For example, many questions that are currently being addressed related to neonatal brain function and development involve short EEG recordings to identify event-related potentials (ERPs) [24, 26–28]. In addition, low numbers of electrodes—as few as one—are often used due to small head size and infant compliance [4, 29–31]. Further challenges also arise because the number of trials in infant studies is often relatively small because infants’ clinical vulnerability limits the number of stimuli which can be applied. In some cases, only a single trial of EEG activity may be available for each participant for ethical or practical reasons. For instance, studies looking at noxious-evoked brain activity often rely on recording the response to a clinically-required stimulus (e.g. a skin-breaking blood test), which is usually only applied once during each recording period [32].

An automated pre-defined artefact detection method that can identify artefacts in short-duration epochs of EEG activity recorded from a single electrode would improve research reproducibility, would reduce reliability on expert raters making EEG a more widely useable tool in neonates, and would be particularly useful in the context of recordings of ERPs in response to sensory stimulation. The aim of this study was to develop an automated artefact detector for ERP studies for neonatal EEG which fulfils these criteria and which was as reliable as a human rater.

## 2. Methods

### 2.1. Datasets and study design

Two internal pre-existing datasets were used to train, validate and test the model. The ‘Oxford multimodal’ dataset consists of 410 unique EEG epochs from 160 infants 28–43 weeks postmenstrual age. These data were collected as part of ongoing studies in John Radcliffe Hospital (Oxford, UK) to investigate infants’ responses to noxious and other sensory stimuli. Each epoch was 1.5 s long and contained one of seven types of event at the 0.5 s mark: auditory, visual, tactile, vibrotactile, heel lance, mild noxious, or no event. This dataset was split into a training set (*n* = 340 epochs), which was used to train and validate the model using cross-validation, and a test set (*n* = 70 epochs), which was used to test the final model on unseen data. This split was stratified by age and stimulus type and grouped by infant. As we envisage that the artefact detection model could be used in clinical trials using neonatal ERPs as an outcome measure, we also tested the model in a clinical trial dataset. The ‘Petal’ dataset consists of 316 unique epochs from 108 infants 35–42 weeks postmenstrual age. These data were collected as part of Petal, a multi-site clinical trial conducted in the John Radcliffe Hospital (Oxford, UK) and the Royal Devon and Exeter Hospital (Exeter, UK) which investigates the impact of parental touch on infants’ responses to a heel lance [33]. Each epoch was 1.5 s long and contained a heel lance, vibrotactile (sham heel lance) or no event at the 0.5 mark.

### 2.2. Data processing (Oxford multimodal dataset)

#### 2.2.1. Participants

Participants were selected from a database of data previously recorded at the John Radcliffe Hospital, Oxford University Hospitals NHS Foundation Trust, Oxford, UK. EEG from 160 infants was selected from that recorded for previous studies, published elsewhere [4, 31, 33–36]. Ethical approval was obtained from the National Research Ethics Service (references: 12/SC/0447, 11/LO/0350 and 19/LO/1085), and informed written parental consent was obtained before each study. Studies conformed to the standards of the Declaration of Helsinki and Good Clinical Practice guidelines.

Infants included were all born between 23–43 weeks gestational age. At the time that EEG recordings were made, infants were aged 28–43 weeks postmenstrual age.

#### 2.2.2. Event types

EEG was recorded in rest and in response to experimental auditory, visual, tactile, vibrotactile and mild noxious stimulation, as well as in response to clinically required heel lances.

Resting state (‘background’) epochs were manually labelled during data collection as periods without external stimulation. Experimental auditory, visual, tactile and noxious stimuli were applied in trains of up to 30 stimuli, with an inter-stimulus interval of at least 10 s which was extended if the infant was unsettled, and automatically time-locked to the EEG. Auditory stimuli of 1 kHz frequency tones were applied using either speakers (X mini MAX II Portable Speakers, Xmi) placed 15 cm from the ears, 100 ms duration at a volume of 80 dB, or using headphones (Surpass Ltd, EMS Biomed), 4 ms duration and a volume of 70 dB nHL. Visual stimuli were single flashes of light (514 lumens, Lifelines Neuro) and tactile stimuli were applied with a modified tendon hammer which was used to gently tap the infant’s heel, and were automatically time-locked to the EEG recording using a force transducer and event detection interface [37]. Experimental mild noxious stimuli (PinPrick, MRC Systems) of 64 mN or 128 mN were applied to the plantar surface of the heel. These were time-locked to the EEG using either an automated trigger (MRC Systems) or a high-speed video recording (220 frames per second [4]).

Heel lances were applied when necessary for blood sampling as part of the infant’s clinical care. Vibrotactile stimuli were applied by performing a ‘control heel lance’, where a heel lance was held against the infant’s foot but rotated by 90 degrees so that when released the blade did not pierce the skin. Both types of event were automatically time-locked to EEG [37].

#### 2.2.3. Data acquisition & processing

Electrophysiological activity was acquired from DC—800 Hz with a sampling rate of 2 kHz using the SynAmps RT 64-channel headbox and amplifiers (Compumedics Neuroscan) and recorded using CURRYscan7 neuroimaging suite (Compumedics Neuroscan). EEG recording electrodes (Ambu Neuroline disposable Ag/AgCl cup electrodes) were positioned on the scalp according to the modified international 10–20 System, with reference and ground electrodes at Fz and the forehead respectively. EEG conductive paste (Elefix EEG paste, Nihon Kodhen) and EEG preparation gel (Nuprep gel, D.O Weaver and Co.) was used to optimise contact and impedances were <10 kΩ. Studies used a varying number of channels, but always included Cz and this is the channel from which we take EEG for this study.

All data analysis was carried out in MATLAB (Mathworks, version R2023a) and Python (Python Software Foundation, version 3.9.12). EEG was band-pass filtered using a non-causal windowed sinc FIR filter to 0.1 Hz–70 Hz [1]. A 49 Hz–51 Hz band-stop filter excluded noise due to mains interference. From each participant, a 1.5 s EEG epoch (−0.5 s to +1 s) was extracted around each stimulus. Where a stimulus was applied to a participant multiple times, only the first stimulus epoch was used in this analysis. This ensured that the effect of inter-subject variation was limited, as each infant contributed only a single EEG epoch for each stimulus type. This gave 495 unique EEG epochs.

The sample of epochs was then balanced for approximately equal numbers of epochs from each stimulus type. Balancing was achieved by removing epochs of types which were overrepresented in the data, and so a trade-off between the balance and size of the dataset was made subjectively. This balancing was completed while blinded to the EEG traces, and the resulting distribution of data across ages and stimulus types is shown in table 1. This gave 410 unique EEG epochs.

**Table 1.** Number of included epochs for each combination of age group and stimulus type. Premature group 1. Infants born prematurely and epoch recorded less than 2 weeks from birth; Premature group 2. Infants born prematurely and epoch recorded more than 2 weeks from birth; term: infants born at term age and epoch recorded less than 2 weeks from birth.

Stimulus type	Premature 1	Premature 2	Term	Total
Background	18	9	31	58
Auditory	14	16	31	61
Visual	13	14	24	51
Tactile	13	13	27	53
Experimental noxious	10	11	39	60
Control lance	18	10	35	63
Heel lance	19	10	35	64
Total	105	83	222	410

#### 2.2.4. Group identification of artefact (ground truth)

To establish a ground truth for artefact, a group of seven human raters independently rated each epoch as either containing artefact or not containing artefact. Epochs were presented in blocks of approximately 100, to reduce rater fatigue, and were randomised within each block. Raters were naïve to stimulus type and infant characteristics, in order to eliminate possible bias from the expectation of stimulus correlated motion. The ground truth (artefact versus no artefact) for a single epoch was determined by majority vote. All raters were paediatric neuroscience researchers from the University of Oxford, UK, who had previous experience with identifying EEG artefact on single channel EEG epochs in a research setting. Three of the group were ‘expert’ raters (with more than 4 years’ experience at EEG artefact detection) and 4 of the group were ‘experienced’ raters (with up to 4 years’ experience).

The instructions supplied to raters were as follows: ‘You are about to be shown 100 epochs of single-channel EEG amplitude data, recorded from term-age and premature infants within 4 weeks of birth. Please review each epoch and decide whether you would reject or include it in an analysis of evoked potential or evoked spectral analysis, based on the presence of artefact. We have included both stimulus-evoked data and data where there is no evoked activity. Where a stimulus has been applied, it is applied at the 0 s time point. You have been chosen because you routinely assess this type of data, and we would like you to treat this as clinical trial data. You will be looking at some short epochs (−0.5 sec to +1.0 sec). The *y*-axis of every plot is scaled to ±150 *µ*V and minimal filtering has been applied (0.1–70 Hz bandpass).’

In order to evaluate the rater performance against the classification performance, Cohen’s Kappa was used to compare the performance of each pair of raters. An individual rater’s average of their Cohen’s Kappa with all other raters is an indication of how well their decisions are aligned with the rest of the group as a whole. This is preferable to using the balanced accuracy as a scoring mechanism, as it takes into account disagreements from the consensus within the group.

#### 2.2.5. Feature selection

To develop a classification model which can identify artefacts in EEG epochs, potential EEG features were identified from existing literature which could predict artefact and which can be calculated for a single, short, single-channel epoch of data. These features are listed below.

• Amplitude change [31, 38]. The maximum amplitude change over any 50 ms period within an epoch.
• Mean local skewness [11, 39]. The absolute local skewness in each 15 ms segment. Here we take the mean of these local skews in an epoch.
• Temporal kurtosis [7, 9]. The kurtosis of each epoch.
• Amplitude variance (maximum local variance) [7]. Here we take the variance of an epoch’s power.
• Derivatives (50/100/200/300 ms)—the sum of derivatives within a window [40]. Here we use as features the maximum absolute sum of derivatives of the amplitude within a window, normalised to the amplitude variance of the epoch. Windows used are 50, 100, 200, 300 and 500 ms, with each producing a separate feature in the model.
• Power <0.5 Hz—the total power below 0.5 Hz [1]. Artefact often manifests at low frequencies, and the previous 0.1 Hz high-pass cut-off allows us to estimate the total low-frequency power in the epoch.
• Frequency band features [11, 39]. These are applied to each EEG frequency band (delta 0.2–3.5 Hz, theta 4–7.5. Hz, alpha 8–13 Hz, beta 14–30 Hz, gamma 30–70 Hz [41]) and also for the whole EEG spectrum—excluding <2 Hz, as EEG spectra tend to only follow a power law above 2 Hz [42]. For each frequency band the following features are calculated.

∗ Band power. This is an estimate of the whole-epoch power spectral density, calculated using a fast Fourier transform.
∗ Lambda. This is the fitted gradient of plot of log signal power to log-frequency, from the equation $P = \lambda {\text{ log}}\left( {{f}} \right) + C$ where *P* is signal power, *f* is frequency, $\lambda $ is lambda, and *C* is a constant [43]. The power is estimated with power spectral density and the fit is approximated using a least-squared curve fit.
∗ Fit error—the maximum spectral deviation. This is the root-mean-square error between the line described by the equation above and the actual signal power spectral density [11].

• Fractal dimension. The fractal dimension of a pattern is the ratio of the change in detail to the change in scale and can be one measure of the complexity of a given signal [44]. It is defined by the equation $N = {\varepsilon ^{ - D}}$ where *N* is the number of measurement units, ***ε*** is the scaling factor and *D* is the fractal dimension. Here we use two alternative estimates of the fractal dimension [45, 46] and the features are the kurtosis of the fractal dimension using each method.
• Wavelets [47, 48]. Wavelet transforms decompose functions into series of waves, giving both spectral and temporal information. The Haar wavelet is one example, and the equation for Haar wavelet *H* at time *t* is shown below, ${\text{H}}\left( t \right) = \left\{ \begin{array}{*{20}{c}} {1{\text{ where}}{\text{ }}0 \unicode{x2A7D} t &lt; 0.5} \\ { - 1{\text{ where}}{\text{ }}0.5 \unicode{x2A7D} t &lt; 1} \end{array}\right.$ Here we calculate the kurtosis of the Haar 1D wavelet transform [49].

Feature selection was performed in the training dataset (*n* = 340 epochs, approximately 6/7 of the available epochs in the Oxford Multimodal Dataset). For each candidate feature, the average absolute magnitude of the feature value for EEG traces classified as clean or artefactual was calculated. The features were plotted in order of *p* value from a binomial logit model comparing that feature’s values for artefact and clean groups.

#### 2.2.6. Model selection and hyperparameter optimisation

Model selection was performed in the training dataset (*n* = 340 epochs, approximately 6/7 of the available epochs in the Oxford Multimodal Dataset, stratified by age and stimulus type and grouped by infant). 71 epochs (21%) in the training set were rated artefact by the majority of raters. Model accuracy was assessed in the training set using leave-one-subject-out cross-validation.

Firstly, the most appropriate supervised-learning classifier was selected. Random forest, support vector machine (SVM), adaptive boosting (AdaBoost), and random under-sampling boosting (RUSBoost) models were evaluated (see supplementary table 1). Performance of the different models was compared using balanced accuracy (the average of sensitivity and specificity), and statistical significance of differences between models was established using McNemar’s Test for paired nominal data. All classifier types performed similarly and none had a statistically significant difference in accuracy. A random forest classifier was therefore chosen for further model development—using scikitlearn v1.0.2 [50]—as these provide an easy and intuitive calculation of the posterior probability and tend to perform well with multiple ‘weak learners’, as is the case with multiple raters making subjective decisions such as this. Posterior probability—the probability that a given epoch would be rejected by the group of raters—is a useful metric for an investigator to review a classifier’s decisions (this is discussed further in the Discussion). Observations were weighted by the certainty of group assessment (which could vary between all 7 raters agreeing and a 3-4 split). A grid search exhaustively searched all hyperparameter combinations using Leave-One-Subject-Out cross-validation. The set of hyperparameters used is shown in supplementary figures 1(a)–(e), with model performance at each hyperparameter value. The grid search selected the set of classifier hyperparameters which gave the best (highest) f1 score weighted for class imbalance. The selected hyperparameters (supplementary table 2) were used to train an artefact identification model using the same weighting and grouping. Leave-One-Subject-Out cross-validation was used to evaluate model performance; confusion matrix data, average Cohen’s Kappa (by treating predictions in the same way as those of a rater) and balanced accuracy were assessed. The ground truth for each rater (including the artefact detector model) was calculated as the consensus decision for all other raters (i.e. each rater’s decisions did not form part of the ground truth estimate for that rater). Balanced accuracy was calculated for each rater from this ground truth.

#### 2.2.7. Performance evaluation in the test set (Oxford multimodal dataset)

Assessment of the performance of the automated artefact detector was carried out on the independent held-out test data from the Oxford Multimodal dataset (*n* = 70 epochs, approximately 1/6 of the Oxford Multimodal dataset). This dataset was kept separate from the training data and was not used for model selection. In the test set, 17 epochs (24%) were rated as artefact by the majority of raters. Confusion matrix data, average Cohen’s Kappa (by treating predictions in the same way as those of a rater) and balanced accuracy were assessed to evaluate model performance.

The ground truth for each rater (including the artefact detector model) was calculated as the consensus decision for all other raters (i.e. each rater’s decisions did not form part of the ground truth estimate for that rater). Balanced accuracy was calculated for each rater from this ground truth.

#### 2.2.8. Performance evaluation in a clinical trial (Petal dataset)

A potential application of the artefact detection model is to use it in a clinical trial which uses ERPs as an outcome measure. Therefore, we additionally tested the artefact detection model in a previously conducted multi-site clinical trial, which involved EEG recordings during a clinically-required heel lance and a control heel lance. This randomised controlled trial [33] investigated the effect of parental touch either before or after routine neonatal blood collection. Details on data acquisition and study design is reported in detail in [33]. As part of the trial, epochs were manually labelled as artefact by two experienced raters. For the purpose of this work, epochs were extracted as for the Oxford Multimodal Dataset, except filtering was performed at 0.5–30 Hz to match the EEG to that assessed by manual raters. Classifier performance was compared to the manual rejections made for the RCT.

In addition to accuracy statistics we calculated the impact of automated artefact identification on the SNR for the clinical trial. A measure of SNR was calculated for each epoch as: $SNR = mag\left( {ERP} \right)/std\left( {baseline} \right)$ [51]. Only epochs with heel lance ERPs were used, for maximum expected ERP magnitude.

#### 2.2.9. Feature importance

Feature importance was assessed in the Oxford Multimodal test dataset and the Petal dataset. For each feature in the trained model, feature importance was calculated by feature permutation (using scikitlearn permutation_importance), randomly permuting each feature 100 times to determine the model’s reliance on each feature. This method was chosen over mean decrease in impurity as it can be computed on any model class, and so better generalises to other models.

#### 2.2.10. Code availability

Original code has been deposited at https://doi.org/10.5281/zenodo.11474626 and is publicly available.

## 3. Results

### 3.1. Artefact in EEG data can be consistently identified by individual raters

**Figure 1.**
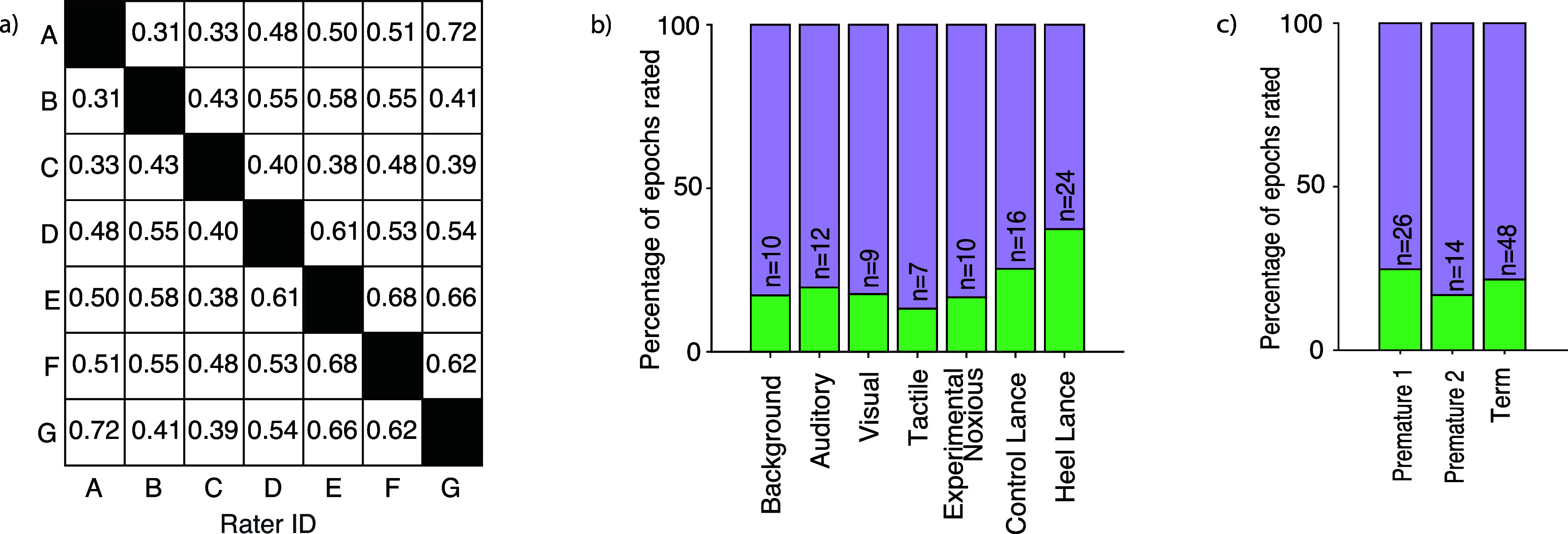
Artefact rating by individuals. (a) Cohen’s Kappa for each pair of raters. Each cell shows the Cohen’s Kappa measure of agreement between a single pair of raters. Raters A–D are ‘experienced’ raters (with up to 4 years’ experience) and E-G are ‘expert’ raters (with more than 4 years’ experience). (b) Percentage of epochs identified as artefact when split by stimulus modality. (c) Percentage of epochs identified as artefact when split by age: premature infants were born less than 37 weeks’ gestation and term were born at 37 weeks’ gestation or above. Premature group 1 are infants who had a postnatal age <2 weeks at the time of study, premature group 2 had a postnatal age >2 weeks, all term-born infants had a postnatal age <2 weeks at the time of study. In these bar charts, the lower (green) bar indicates epochs rated as artefact, while the higher (purple) bar indicates epochs rated as clean. Numbers on bars (i.e. *n* = *X*) indicate the absolute number of epochs represented by the green bar (i.e. the number of epochs containing artefact).

Seven human raters independently labelled a training dataset that contained 340 1.5-second epochs of EEG activity according to whether or not they contained artefacts. The EEG data included background activity (where no stimulus was applied) and responses to visual, auditory, tactile and noxious stimuli. The agreement between individual raters was ‘fair to good’, indicated by an average Cohen’s Kappa of 0.51 (figure 1(a)) [52]. The proportion of epochs rated as artefactual did not substantially differ across stimulus types (figure 1(b)) or age groups (figure 1(c)). As expected, agreement between raters related to their level of experience—Cohen’s kappa between ‘expert’ raters (>4 years’ experience) was 0.61 compared with 0.40 between ‘experienced’ raters (1–4 years’ experience working with EEG, figure 1(a)).

### 3.2. EEG features are altered by artefact

**Figure 2.**
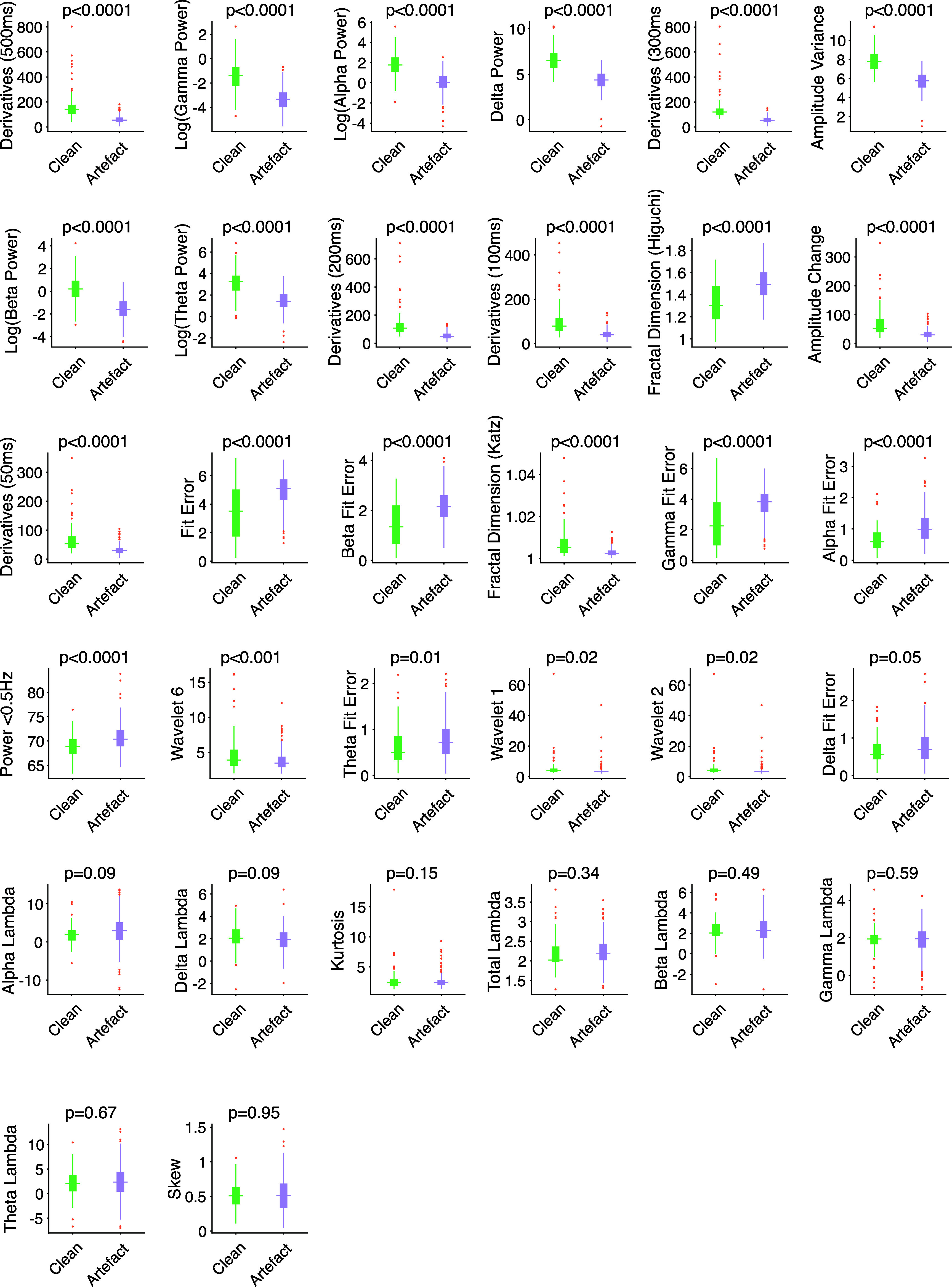
The magnitude of the absolute feature value in clean and artefactual epochs of EEG activity (as decided by majority decision from raters). Above each plot is the p value related to the null hypothesis that the data are drawn from the same population (as calculated by binomial logit model for that feature). On each plot, the box indicates the range of the 25th and 75th percentiles of the absolute feature magnitude. The line central in the box indicates the median. The whiskers extend to the most extreme non-outlier values. Points indicate outliers, assessed as values more than 2.7 standard deviations from the mean. Feature values are transformed into log values where appropriate for easier viewing—as shown in y-axis labels.

A set of candidate EEG features that could be used to classify whether an epoch was considered to contain artefacts were identified from the literature (see Methods). This included time-domain features (e.g. voltage amplitude variance, sum of derivatives within a window), frequency-domain features (e.g. frequency band power, maximum spectral deviation), and features using both the time and frequency domain (e.g. wavelet transform decompositions). For each candidate feature, the absolute magnitude of the feature value for EEG traces classified as clean or artefactual is shown in figure 2. This shows the features with the best potential for predicting artefact, although all features potentially contribute something to a combined classifier, even if not statistically significant by themselves. No features were significantly associated with age (supplementary figure 2). All features were used as part of the classification algorithm.

### 3.3. Artefact detection can be automated using a machine learning classifier

**Figure 3.**
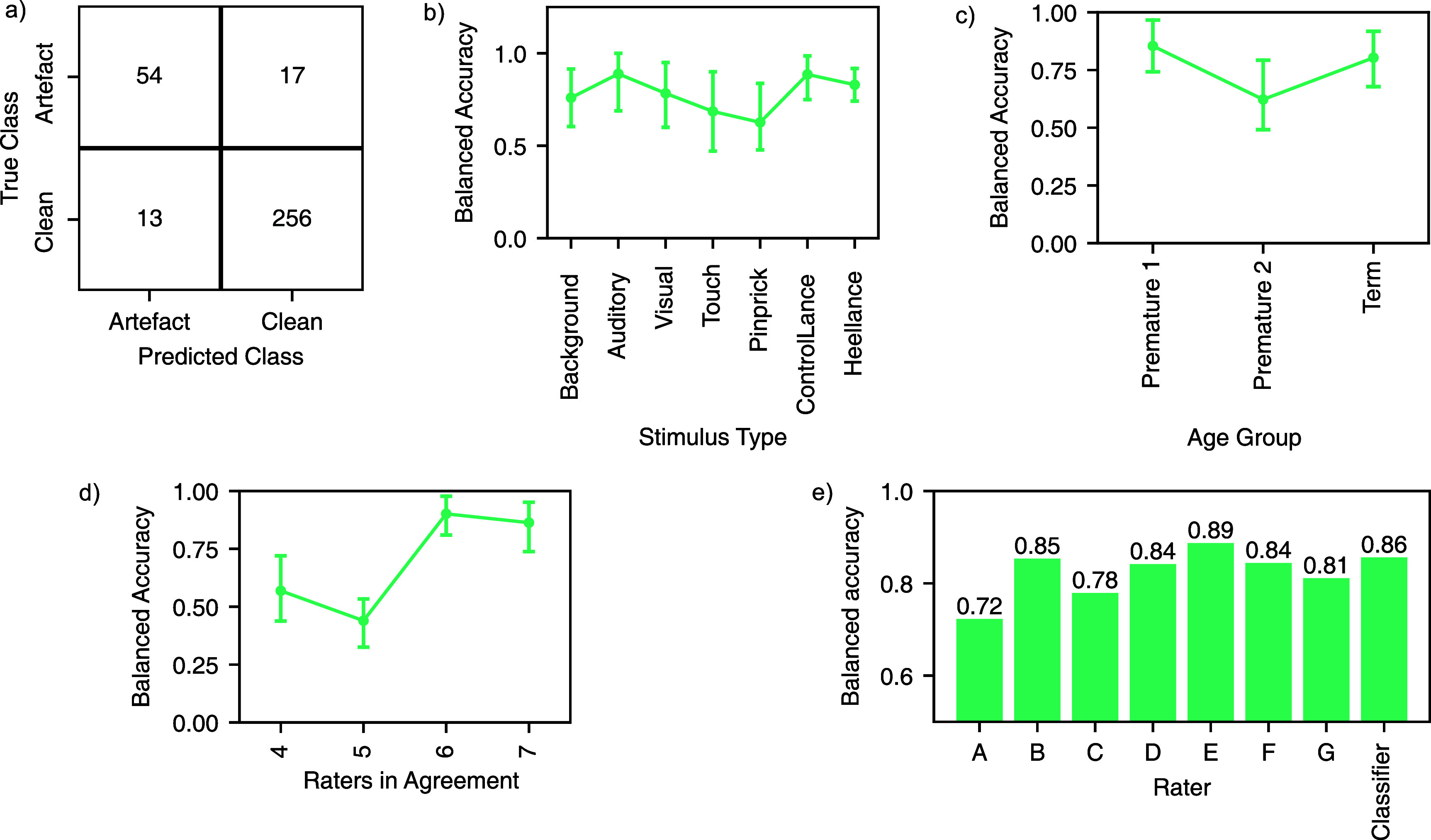
Model performance for the automated artefact detector, assessed using five-fold cross-validation in the training set. Validation folds were stratified by age and stimulus type and grouped by infant. (a) Confusion matrix for data in the training set (*n* = 340). Numbers indicate the number of epochs classified correctly and incorrectly according to whether or not the epoch contained artefact. True class indicates the majority decision of the raters, predicted class indicates the class predicted by the automated artefact detector. From this the sensitivity (0.76), specificity (0.95) and balanced accuracy (0.86) can be derived. (b) Classifier balanced accuracy according to type of stimulation in each EEG epoch—error bars indicate 95% bootstrapped confidence interval; (c) classifier balanced accuracy according to age group, where the group ‘premature 1’ is infants born prematurely with postnatal age less than 2 weeks at time of test, and ‘premature 2’ is infants born prematurely with postnatal age 2 weeks or above at time of test—error bars indicate 95% bootstrapped confidence interval; (d) classifier performance on subsets of the data split according to the number of raters in agreement with the consensus—error bars indicate 95% bootstrapped confidence interval; (e) balanced accuracy, calculated using leave-one-rater-out for each rater. The classifier has a good performance compared to both experienced (A–D) and expert raters (E–G).

A classification model was created to identify epochs of EEG activity with artefact compared to those without artefact (as defined by the consensus rater agreement), with four different models compared (see Methods). All models tested were comparable in terms of model performance (*p* > 0.05, supplementary table 1). The random forest model was used for subsequent analysis as the ensemble method used in a random forest allows for an easy and intuitive calculation of the posterior probability. This random forest model achieved a balanced accuracy of 0.86, a sensitivity of 0.80 and a specificity of 0.93 (assessed using cross-validation) in the training dataset (figure 3(a)). Model performance was similar across different stimulus types (figure 3(b)) and different age groups (figure 3(c)).

The performance of the artefact detection classifier was better for those epochs with high rater agreement (figure 3(d)). For example, when 6 of the 7 raters were consistent in the classification of the epoch the balanced accuracy was 0.95, the sensitivity 0.90, and the specificity 0.99 (*n* = 254 epochs). When there was no clear consensus among raters (i.e. when only 4 out of 7 raters reached a majority agreement) the model performance was lower (balanced accuracy = 0.68; sensitivity = 0.55; specificity = 0.81; *n* = 64 epochs). This is as expected—those epochs where all raters agreed are likely to have overt artefacts more easily detectable, whereas epochs where only 4 out of 7 raters classed the epoch in one way (and the other 3 gave the opposite classification) are likely to have less obvious distinguishing features. Similarly, in random forest models, each individual decision tree produces a predicted class probability and the mean of all trees gives the posterior probability for a given class label. As expected, higher posterior probabilities were generally correlated with higher rater agreement, and the curve approximates a logistic function (supplementary figure 3).

When measuring agreement with manual raters, the artefact detection classifier had an average Cohen’s Kappa of 0.57, which compared favourably with the consensus rater assessment. Figure 3(e) shows that the balanced accuracy of the classifier was comparable to that achieved by the individual raters, and overall had greater accuracy than 6 of the 7 individual raters. It performed better than the average balanced accuracy of both the experienced raters (0.80) and the expert raters (0.85).

### 3.4. Application of the artefact detection algorithm in an independent held-out dataset

**Figure 4.**
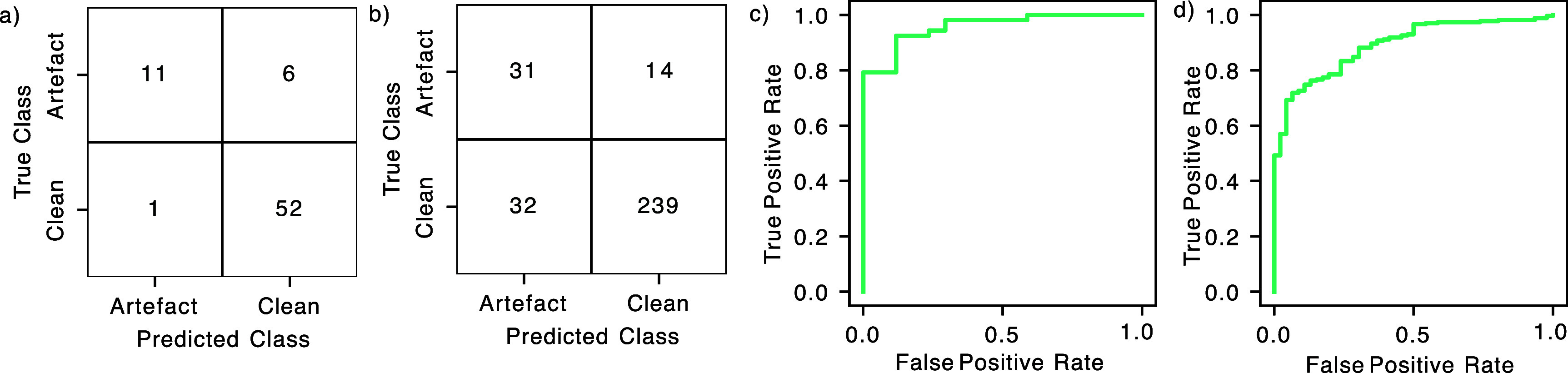
Testing the artefact detector in independent test sets. (a) Confusion matrix showing performance of the artefact detector on novel data from the held-out test dataset: 0.81 balanced accuracy, 0.65 sensitivity, 0.98 specificity. (b) Confusion matrix showing performance of the artefact detector on novel data from the Petal clinical trial: 0.78 balanced accuracy, 0.67 sensitivity, 0.88 specificity. (c) Receiver operator characteristic (ROC) curve, when tested on the held-out test dataset. Area under the curve: 0.96. (d) Receiver operator characteristic (ROC) curve, when tested on novel data from a clinical trial. Area under the curve: 0.89.

To confirm the cross-validation results and estimate the prediction accuracy of the artefact detection classifier on unseen data, the classifier was applied to an independent held-out dataset. When tested on 70 held-out EEG epochs, the model had a balanced accuracy of 0.81 (sensitivity 0.65, specificity 0.98; figure 4(a)). This compares well to an average balanced accuracy of 0.84 for all raters (calculated using the same leave-one-rater-out). Similar to the training data, comparison of posterior probability with rater agreement shows an approximation of a logistic function, suggesting that epochs which were easy for manual raters to classify were also easy for the detection algorithm to classify (supplementary figure 3).

The classifier is optimised for maximum balanced accuracy, and the results given here are for that optimisation. It may be desirable to use a different balance of sensitivity and specificity: for example to prioritise a low false-negative rate. For this we can vary the probability threshold at which an epoch is classified as clean or artefact. The receiver operating characteristic (ROC) curve shows how false positive rate and true positive rate vary as the threshold is varied (figure 4(c)).

**Figure 5.**
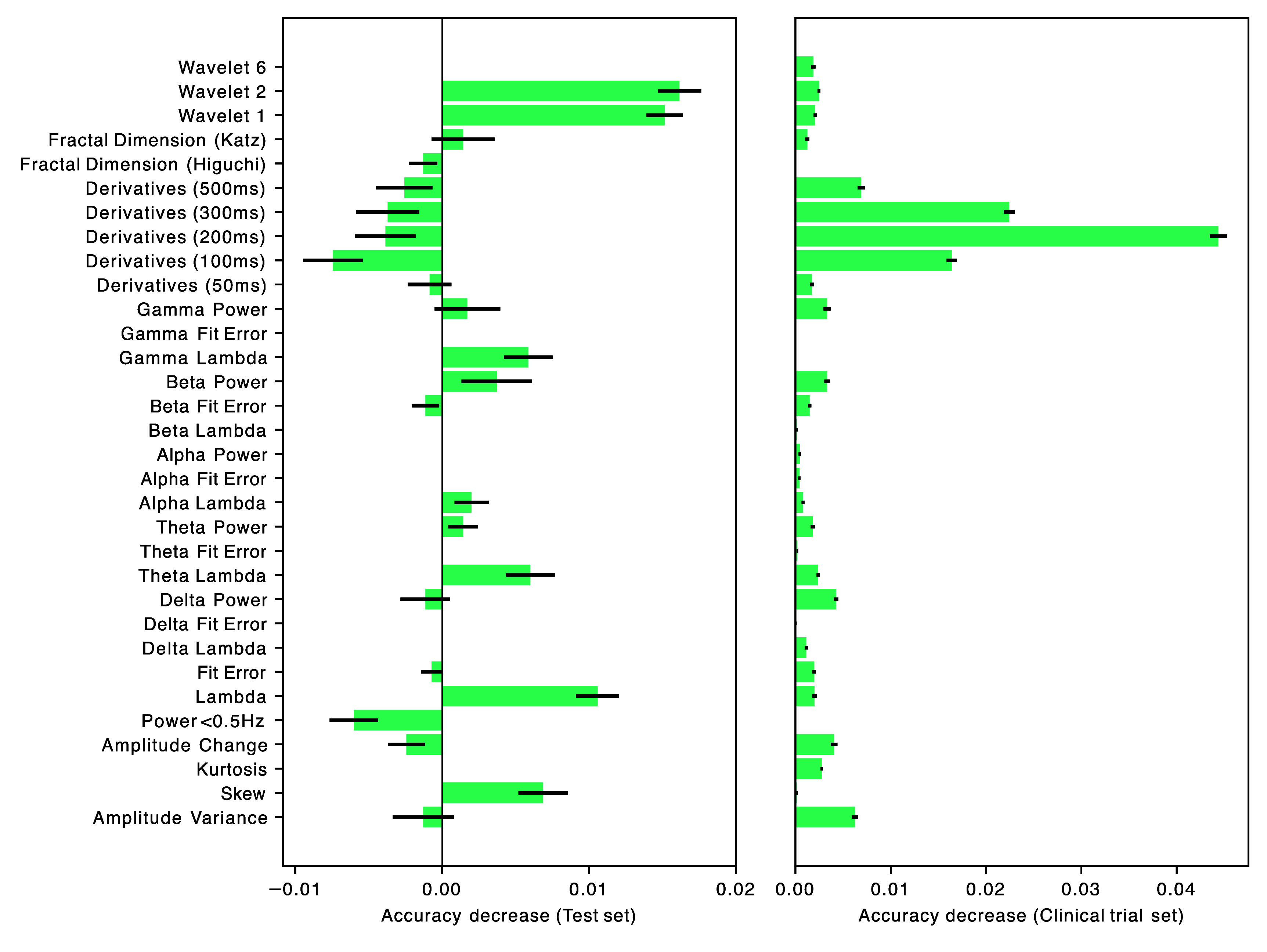
Feature importance when calculated on the held-out test dataset (left) and on the clinical trial dataset (right). For each feature in the trained model, feature importance was calculated by feature permutation (using scikitlearn permutation_importance). For each feature this randomly permutes the relationship between feature value and epoch 100 times. The decrease in the model’s accuracy when a feature is permuted is a measure of the importance of that feature. For each feature, the values shown here are for the mean and 95% Confidence Interval of the decrease in model accuracy for those 100 permutations. The importance of features with no bar was unable to be identified.

Feature importance for this dataset is shown in figure 5 (left).

### 3.5. Application of the artefact detection algorithm in a clinical trial

The model is designed to provide an unbiased approach to rejecting data based on the presence of artefacts, and the previous test shows performance on held-out data from the same dataset. A method’s ability to generalise to other data is particularly useful in the context of a clinical trial and so we also assessed performance on a second independent test set that was collected as part of a clinical trial. To assess its use in this context, the artefact detection classifier was applied to epochs of EEG collected in the Petal clinical trial [33]. In the Petal trial, artefact was identified based on the consensus decision of two expert raters. Of the 316 epochs of data included in the trial (104 noxious heel lance epochs, 105 non-noxious control heel lance epochs and 107 background epochs with no stimulus), manual identification of artefacts in the original study led to 46 epochs being identified as artefactual. In comparison, when the automated artefact detection classifier was used, 103 data epochs were identified as artefactual. A confusion matrix shows the performance of the artefact detector on the novel clinical trial data: 0.78 balanced accuracy, 0.67 sensitivity, 0.88 specificity (figure 4(b)).

The ROC curve (figure 4(d)) has an area under the curve of 0.89. This is similar to, and slightly lower than, that of the test set, which has an area under the curve of 0.96.

The mean SNR was calculated for the group of heel lance epochs identified as clean by the detector (*n* = 68, SNR = 0.052), and for the group of heel lance epochs included in the original clinical trial (*n* = 82, SNR = 0.046). SNR using the automated method was 12% higher than using the expert team of the original trial.

Feature importance for this dataset is shown in figure 5 (right), alongside that of the held-out dataset.

## 4. Discussion

We have created an automated artefact detector for stimulus-evoked EEG data in infants and demonstrated that it performs well on single-subject data with low numbers of channels and short epochs. Accuracy is as good as manual review of data by individual raters, and it has the benefits of improved reproducibility, speed of analysis and could be used by non-expert users. Use of this method has benefits over those used by recent publications which present artefact identification algorithms [19–21], as it can be used on very short, single-channel epochs.

Current methods for artefact identification in neonatal EEG are not ideal. A number of publications in the field involve the analysis of short epochs of low- or single-channel EEG data recorded from young infants, and most-commonly state that a manual review method is used to exclude artefactual data. Many publications do not report the methods used for artefact detection and exclusion, and often simply state the number of trials that were excluded (Fabrizi *et al* [24], Gursul *et al* [55], Norman *et al* [26], van der Vaart *et al* [36]). Slater *et al* [32] used a 50 *µ*V/50 ms (in >15 electrodes) ceiling, rejecting anything above that as movement artefact. Maimon *et al* [25] viewed 30-second increments of the average signal of Fp1, Fp2, Pz, C1, C2 and accepted anything ‘free of movements and electrical induction artefact’. In Schmidt Mellado *et al* [31] a neurophysiologist excluded individual epochs ‘if the pre-stimulus baseline or the post-stimulus activity contained gross movement artefacts (e.g. signal amplitudes >800 *µ*V on any channel), or if the pre-stimulus baseline was unsettled (i.e. when stimulation was preceded by spontaneous bursting or other activity in the background or when EEG activity prior to stimulus was fluctuating >20 *µ*V)’. These examples highlight the range of different approaches that have been reported.

Neonatal ERPs can be used as outcome measures for clinical trials [32, 33, 53, 54]. Artefact can contaminate EEG recordings and thus the precision with which treatment effects can be identified is reduced, as it impacts the SNR in the data. By identifying and removing trials contaminated by artefact, the SNR and the precision with which treatment effects can be identified can be improved. Importantly, by performing this identification using an automated classifier we can do this reproducibly across studies. This study shows that the proposed model generalises well to other ERP datasets and so it has the potential to be used in future clinical trials that use ERP data.

Our results show that the proposed method of automated artefact detection performs approximately as well as manual raters when applied to novel data. It performs well across a number of different types of stimuli, and across different neonatal age groups. Both posterior probability and accuracy are related to agreement between manual raters, suggesting that epochs which are clearly considered to be artefactual or not by manual raters are also highly likely to be correctly classified using the automated method. In addition, the automated method is performed very quickly (for a single sample, 477.55 ms ± 12.00 ms for feature calculation and 7.76 ms ± 0.06 ms for classification).

One potential way of using this automated artefact detector is to use the automated method as a first review pass, and for the investigator to then review the segments of EEG data, together with the automated method’s decisions and posterior probability, in order to confirm or dispute those decisions. Similar approaches are commonly used in in the labelling of artefactual EEG components obtained through ICA [15]. The investigator could then record those epochs for which the automated method’s decision was disputed. This improves experimental reproducibility over existing methods. In addition, compared to a single manual rater, this method should improve the accuracy of artefact identification and it could also be used as a training tool.

Interestingly, epochs labelled as artefact had on average a lower amplitude variance and measures correlated with it (such as sums of derivatives). This is unusual because EEG with artefact often has higher absolute signal magnitudes and variances [14]. As most epochs in this dataset contain ERPs, it is possible that some artefacts—such as electrode detachment—reduced the variance from ERPs. Alternatively this dataset may contain more low-variance artefacts such as skin-gel interface resistance or electrode detachment, and fewer high-variance artefacts such as blink, movement or ECG breakthrough. However, this study did not classify artefacts by type.

Model performance—assessed using leave-one-subject-out cross-validation—was not very sensitive to hyperparameter selection (supplementary figures 1(a)–(e)). Samples per leaf (supplementary figure 1(c)) was the only hyperparameter whose optimisation visibly improved performance. Lastly, permutation feature importance testing suggests that not all features add predictive value to the model (figure 5) even though all features appeared to have some relation to the presence of artefact (figure 2). Permutation feature importance can attach low importance values to features which are highly correlated with one another. This is because when a correlated feature is permuted, the model still has access to information about it through its correlated features. Most of the features used in the model were correlated with one another to some extent and so it is possible that permutation feature importance testing is under-estimating feature importance in correlated features.

There are some limitations to this analysis. In particular, the relatively small size and imbalanced classes in the datasets used (with artefact being less prevalent than non-artefact observations) may limit the performance of the model. We reduced the impact of this by grouping and stratifying the training data in order to reduce over-fitting. Balanced accuracy on the held-out dataset (0.81) was similar to the balanced accuracy of the training dataset (0.86) which suggests that the impact of over-fitting was small. In the future, it is possible that performance may be improved with the use of additional training data. Importantly, this method also performs well on data recorded as part of a clinical trial and on individual measurements as would be recorded in a clinical setting and does not require subject-specific adaptation. This makes it potentially useful in clinical applications, where data from a single individual is considered.

Different applications have different requirements for artefact classification, and for some applications the probability threshold presented here is not appropriate. In some clinical uses a high specificity may be required in order to be as certain as possible that a clinical decision is not made based on data contaminated by artefact. On the other hand, use in a clinical trial may prefer to minimise the amount of data which is rejected in order to minimise the possibility of bias. For example, the classifier incorrectly classified as artefact 68 epochs from the Petal clinical trial, 21.7% of the included epochs (figure 4(b)), and this would result in a large rate of data rejection in the trial. In order to adjust the classifier for different priorities, a user can adjust the probability threshold at which an epoch is classified as artefact. The effect of this is shown in figures 4(c) and (d). In this way a sensitivity-specificity balance can be chosen for a particular application.

This classifier has been designed specifically for use in single-channel EEG data. Hypothetically the classifier can be applied to any number of channels (it is not channel-specific), but this has not been validated here. In particular, manual rating of artefact in multi-channel EEG often relies on inter-channel information (e.g. in identifying ‘break-through’) and many existing automated methods use channel covariance information. This limitation is not a design flaw, but a result of the design decision to focus on single-channel data. The method which we present here neglects inter-channel data by design.

In conclusion, we created and tested an automated method of detecting artefact in individual, single-channel epochs of infant EEG. It fills a niche in the automated identification of artefact in low-channel ERP analysis. This method has the benefits of reproducibility and speed of analysis when compared to the manual methods that are commonly used.

## Data Availability

The data cannot be made publicly available upon publication because they contain sensitive personal information. The data that support the findings of this study are available upon reasonable request from the authors.
